# Design and Evaluation of a Non-Contact Bed-Mounted Sensing Device for Automated In-Home Detection of Obstructive Sleep Apnea: A Pilot Study

**DOI:** 10.3390/bios9030090

**Published:** 2019-07-22

**Authors:** Clara Mosquera-Lopez, Joseph Leitschuh, John Condon, Chad C. Hagen, Uma Rajhbeharrysingh, Cody Hanks, Peter G. Jacobs

**Affiliations:** 1 Artificial Intelligence for Medical Systems (AIMS) Lab, Department of Biomedical Engineering, Oregon Health and Science University, Portland, OR 97239, USA; 2 Proto-tech Research, Portland, OR 97267, USA; 3 Department of Electrical and Computer Engineering, Portland State University, Portland, OR 97201, USA

**Keywords:** automated obstructive sleep apnea diagnosis, unobtrusive obstructive sleep apnea diagnosis, Apnea-Hypopnea Index, contact-less load cell sensor, long-term breathing monitoring

## Abstract

We conducted a pilot study to evaluate the accuracy of a custom built non-contact pressure-sensitive device in diagnosing obstructive sleep apnea (OSA) severity as an alternative to in-laboratory polysomnography (PSG) and a Type 3 in-home sleep apnea test (HSAT). Fourteen patients completed PSG sleep studies for one night with simultaneous recording from our load-cell-based sensing device in the bed. Subjects subsequently installed pressure sensors in their bed at home and recorded signals for up to four nights. Machine learning models were optimized to classify sleep apnea severity using a standardized American Academy of Sleep Medicine (AASM) scoring of the gold standard studies as reference. On a per-night basis, our model reached a correct OSA detection rate of 82.9% (sensitivity = 88.9%, specificity = 76.5%), and OSA severity classification accuracy of 74.3% (61.5% and 81.8% correctly classified in-clinic and in-home tests, respectively). There was no difference in Apnea Hypopnea Index (AHI) estimation when subjects wore HSAT sensors versus load cells (LCs) only (*p*-value = 0.62). Our in-home diagnostic system provides an unobtrusive method for detecting OSA with high sensitivity and may potentially be used for long-term monitoring of breathing during sleep. Further research is needed to address the lower specificity resulting from using the highest AHI from repeated samples.

## 1. Introduction

Obstructive Sleep Apnea (OSA) is a prevalent medical condition that occurs when the upper airway becomes blocked repeatedly during sleep causing breathing to slow or completely stop. It is estimated that approximately 15% of the population suffers from OSA [1,2], but despite its prevalence many clinically significant cases remain undiagnosed [3,4]. The gold standard for a definitive diagnosis of OSA is overnight polysomnography (PSG) testing [5]. PSG is carried out at a sleep clinic where patients spend the night with multiple sensors attached to their body to measure physiologic metrics, including airflow, respiratory effort, blood oxygen saturation levels, electrical brain activity, eye movement, muscle activity, electrical heart activity, and snoring. These signals are analyzed by a trained clinician in order to detect the presence and duration of apnea and hypopnea events. However, there is a scarcity of specialized clinics and trained professionals that are available to diagnose OSA, which leads to a significant percentage of people with OSA who are undiagnosed and thus untreated [2]. Furthermore, PSG is an obtrusive sleep test that can directly interfere with sleep. This negative impact on sleep can lead to the well-known “first night effect” [6], whereby the sleep apnea detected on the first night of PSG measurements is different than on subsequent nights as the patient gets accustomed to the sensors.

Recently, in-home sleep apnea testing (HSAT) has become more common. HSAT uses a reduced set of sensors that are worn on the face and chest of the patient for use at home. However, the sensors used within HSAT, while less invasive than PSG, are still obtrusive and may interfere with a person’s normal sleep. Furthermore, people who use HSAT are required to monitor and sustain the adequate position and contact of sensors to obtain useful diagnostic data. People do not recognize sensor loss while asleep and may not notice when awake in order to address signal loss, which can lead to failed diagnoses.

OSA severity, detected using either PSG or HSAT, is measured using the Apnea Hypopnea Index (AHI), which is the average number of apnea and hypopnea events measured over the total sleep time. Clinically, an AHI value less than five events/h is considered normal as supported by epidemiological evidence that showed a minimal impact on health outcomes for this many events per h. An AHI of 5–14 events/h is classified as mild sleep apnea, 15–29 events/h is moderate, while an AHI greater than 29 events/h is severe [5]. Patients with mild apnea have a wider variety of options, and the course of treatment depends on the presence of OSA related symptoms, including sleepiness, impaired cognition, mood disorders, or cardiovascular morbidity. However, for patients with moderate-to-severe OSA, corresponding to an AHI > 15 events/h, nasal continuous positive airway pressure (CPAP) is often the gold standard treatment [7,8,9,10].

Various research groups have developed sleep monitoring and diagnostic devices that are both unobtrusive and accurate. For instance, Brink et al. [11] employed load cells (LCs) to measure basic sleep physiology parameters, including heart rate, respiration rate, and body movements, during sleep. Austin et al. [12] and Beattie et al. [13,14] studied the use of LCs placed under the supports of a bed to distinguish periods of sleep and wakefulness (with sensitivity of 0.808 and specificity of 0.812), detect respiration signals and measure its rate (with mean error of 0.18 breaths/min), and identify clinically relevant breathing disturbances, such as apneas and hypopneas. In recent work published by Beattie et al. [15], the main objective was to evaluate the correlation between manually scored PSG and LC sleep study scores, which was found to be 0.97. The reported OSA detection sensitivity for a cutoff AHI > 5 events/h was 84.0%.

Ultrasound technologies have also been used for capturing breathing signals and for diagnosing sleep disordered breathing conditions as an alternative to obtrusive PSG and HSAT tests. Arlotto et al. [16] designed a low-power ultrasonic Doppler device that measures the frequency shift produced by the velocity difference between the exhaled airflow and the ambient environment and accurately captures a breathing signal that is analyzed to detect changes in its intensity and rate. However, to the best of our knowledge, this approach has not been clinically tested on multiple subjects.

While there are a plethora of smartphone based applications which claim to detect sleep apnea, many of these have not yet been clinically evaluated [17]. Nandakumar et al. [18] used ultrasound emitted from the speaker of a smartphone and measured by the onboard microphone to capture chest movement to classify AHI severity achieving an average error of 1.9 events/h.

In the current study, we assess the diagnostic accuracy of a novel AHI measurement system designed in our laboratory at Oregon Health and Science University (OHSU) [19]. We present accuracy results obtained both within a monitored sleep clinic and under free living conditions in patients’ homes. The system is comprised of an array of multiple LCs arranged on a rectangular metallic plate that is placed underneath the patient’s mattress. The system includes signal processing and machine learning algorithms that are able to (1) extract the relevant breathing and movement-related signals and (2) process these signals with a decision tree and linear regression algorithm to classify OSA severity. Previously developed LC-based monitoring systems with LCs positioned under the bed or integrated in the bedframe require installation by a trained technician. In contrast, our system is easily deployable at home by the patient. Moreover, we automatically process LC signals to diagnose OSA, so that manual scoring by a technician is not required. We show the performance of our sensing device across data collected in the sleep lab when patients wore PSG sensors, in-home data when patients wore HSAT sensors, and also in-home data when patients did not wear HSAT sensors when presumably we would capture more of a typical night of sleep.

This paper makes several contributions beyond what we had previously published [19]. First, we present the analysis of the accuracy of our sensing device against gold standard PSG and HSAT tests, which shows that our sensing system and the proposed automated signal processing pipeline achieved high sensitivity in detecting the presence and severity of OSA in-clinic and in-home settings. Second, we evaluate the effect of wearing HSAT sensors on the AHI estimated by our system, where we found that there was no statically significant difference between the average AHI during the nights the participants wore HSAT sensors versus the nights when they did not wear additional HSAT sensors. Finally, we present the results of a usability survey that allow us to demonstrate the deployability of the systems in unattended home settings in terms of ease of installation and effect on bed stability and comfort during the home sleep test.

## 2. Materials and Methods

### 2.1. Cohort and Sleep Studies

Fourteen subjects (11 females and 3 males) from the OHSU sleep clinic were recruited to participate in a pilot study in order to evaluate our sensing device in terms of its accuracy in estimating AHI and diagnosing OSA severity. All subjects gave their informed consent for inclusion before they participated in the study. The protocol of the study was approved by the OHSU institutional review board (IRB), under project identification code IRB00006308 and conducted in accordance with OHSU IRB policies and procedures.

**Figure 1:**
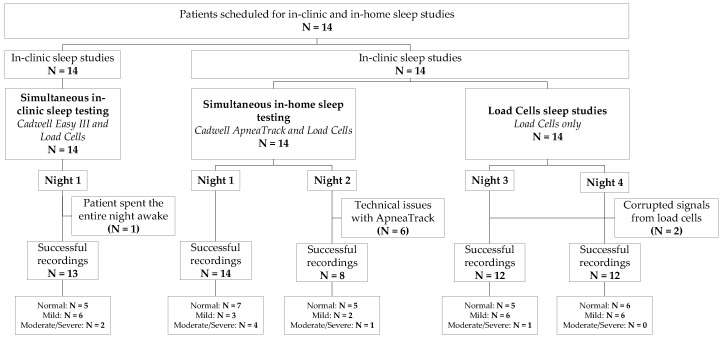
Flow chart of patients who underwent sleep studies.

The average age was 48 ± 21 years and the average body mass index was 33.49 ± 7.83 Kg/m^2^. All participants were scheduled for in-clinic overnight diagnostic PSG, two consecutive nights of in-home testing with HSAT sensors, and two consecutive nights of in-home sleep apnea testing with no HSAT sensors. Figure 1 presents a flow chart of subjects who underwent sleep studies. Physiological signals of interest were acquired and recorded using Cadwell Easy III PSG and ApneaTrak HSAT systems (Cadwell, Kennewick, WA, USA) during in-clinic and in-home sleep studies, respectively. While the study was designed to collect two nights of HSAT data per subject, technical problems with the ApneaTrak arose for various reasons and for some of the subjects, only one night of in-home HSAT data were collected. LC sensor signals were simultaneously recorded during every night of the study using an array of contact-free LCs located underneath the patient’s mattress. PSG and HSAT studies were scored by a registered polysomnographic technologist (RPSGT) expert and blindly reviewed by a board certified sleep specialist according to American Academy of Sleep Medicine (AASM) scoring guidelines [20]. The AASM recommended hypopnea scoring rule requiring a 3% desaturation or arousal for inclusion of hypopnea was used. The calculated AHI was then used as ground truth to optimize machine learning models for automated detection of OSA and classification of the disease severity.

Participants were given a brief survey at the conclusion of the in-home portion of the study, whereby they were asked how easy it was to install the system, (2) how stable the bed felt while sleeping with the system installed, and (3) whether the bed felt comfortable while they were sleeping with the LC system installed. The survey was scored on a 1–5 basis, whereby 5 was the best score.

### 2.2. Data Acquisition System

**Figure 2:**
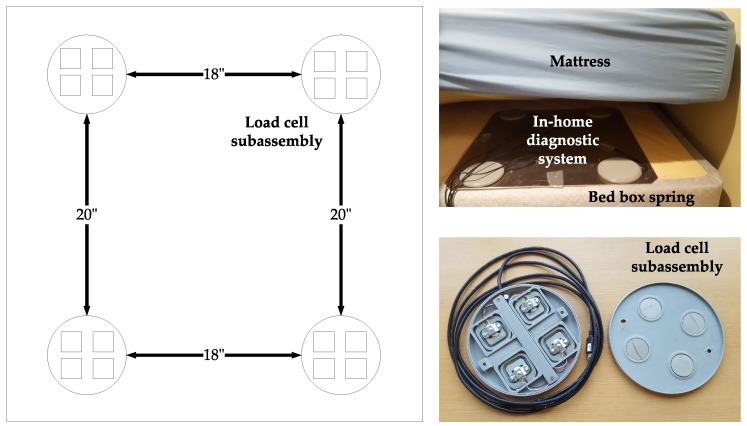
Oregon Health and Science University (OHSU)’s custom built data acquisition system.

**Figure 3:**
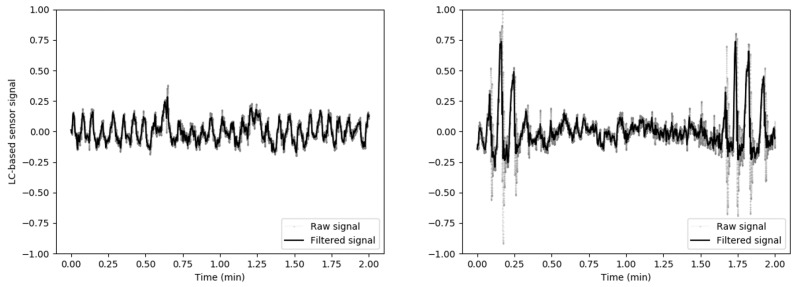
Examples of signals obtained from our load cell (LC)-based sensing device.

A novel contact-free sensing device, located underneath the patient’s mattress, developed in our laboratory at OHSU was employed to capture breathing and movement signals at a sampling rate of 250 Hz (see Figure 2). Simultaneously, data were collected from PSG and HSAT sensors (nasal cannula and abdomen belt were employed) attached to the patient’s body. Our sleep monitoring device was built by integrating ultra-low profile LCs (Detail-Tec, Changsha, China) into four custom 3D printed enclosure subassemblies affixed in a rectangular metallic plate. The distribution of the subassemblies within the aluminum plate was such that two cells would register the weight on either side of the body across the shoulders and the other two cells would target the weight in the hip. A more detailed description of the design and fabrication of the LC-based acquisition system was provided in our previously published work [19]. The data collected from the LCs was relayed to an Odroid-U3 processor (Samsung Electronics Co. Ltd., Suwon, Korea) via a serial peripheral interface (SPI) bus. The Odroid formatted the data and then sent it to a Google Drive (Google, Mountain View, CA, USA) cloud location for offline processing. Scientific libraries for Python 3.6.3, including Numpy, Scipy, Scikit-learn, Pandas, and Matplotlib, were employed for signal processing and training of machine learning algorithms on a Linux environment. Figure 3 presents an example of the signals obtained from our device. In its current, our LC-based system is not designed to identify individual apnea or hypopnea events, but the signals are processed to make it possible to estimate AHI, which is ultimately employed to classify OSA severity in clinical practice.

### 2.3. Load Cells Signal Processing and Feature Extraction

Raw pressure signals from each of the four LC subassemblies were combined by adding time-aligned samples from the four subassemblies. The resulting signal was then processed using a band-pass filter to eliminate low frequencies corresponding to signal drift and high frequencies greater than 5 Hz corresponding to non-physiologic noise harmonics. Next, the resulting signal was scaled to have zero mean and unit standard deviation. The Fast Fourier Transform (FFT) was used to calculate the power spectral density (PSD) of the processed LC signal and statistical moments within consecutive non-overlapping frequency sub-bands of 0.02 Hz width were computed in order to characterize the distribution of the magnitude of Fourier coefficients [19].

**Figure 4:**
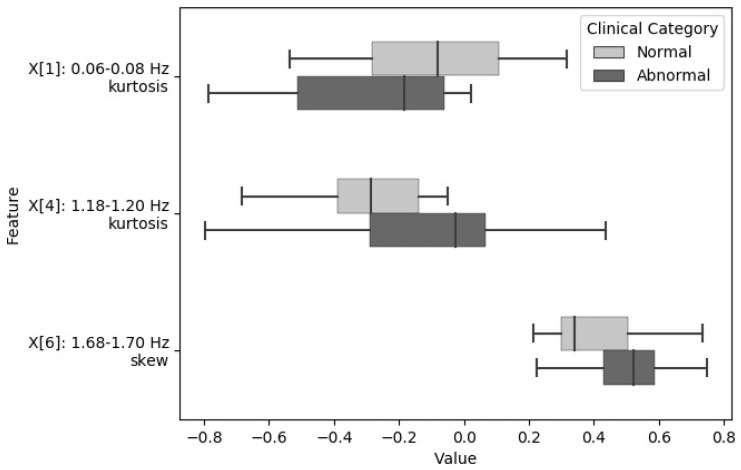
Statistical distribution of spectral features used for obstructive sleep apnea (OSA) detection grouped by clinical category.

**Figure 5:**
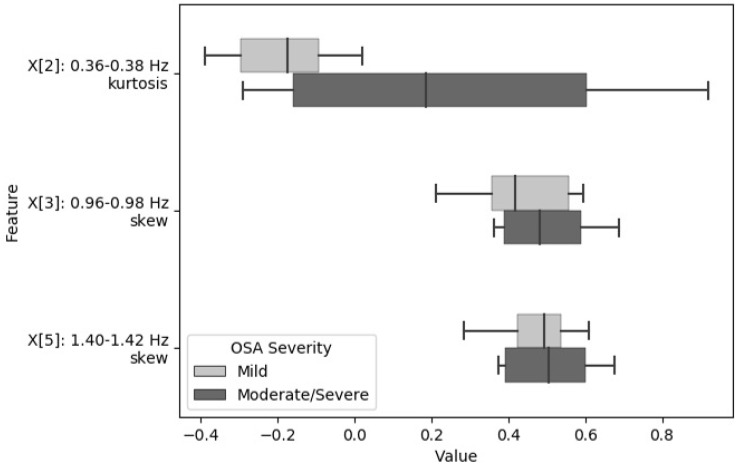
Statistical distribution of spectral features used for Apnea Hypopnea Index (AHI) estimation grouped by clinical OSA severity.

Using analysis of variance (ANOVA) and F-test, the skewness and kurtosis from six frequency sub-bands were selected as the most relevant descriptors to be used to model the severity of OSA using all the available data. These features had the highest F-score when analyzed against the target severity categories (normal, mild, and moderate/severe). The selected frequency bands are as follows: 0.06–0.08 Hz, 0.36–0.38 Hz, 0.96–0.98 Hz, 1.18–1.20 Hz, 1.40–1.42 Hz, and 1.68–1.70 Hz (Mosquera-Lopez et al., 2018). Figure 4 and Figure 5 show the distribution of features extracted from relevant frequency sub-bands grouped by OSA severity. The box plots show the median value of the features for all subjects within a given OSA severity category, as well as the interquartile range.

### 2.4. Machine Learning Models

We developed a two-stage classifier to automatically detect OSA and classify its severity. Our previous work [19] discusses the architecture of the classification system. In order to assess the generalization performance of the developed algorithms, we used leave-one-patient-out cross-validation. That is, all data collected for a given subject over multiple nights irrespective of the gold standard sleep test were held out from the training set when predicting OSA severity for the subject. This validation strategy is advantageous when the dataset size is small because the bias is reduced as each cross-validated model is trained almost on the entire dataset.

Our classification method, trained using spectral features presented in Table 1, is comprised of two models: (1) stage-one decision tree (S1DT) and (2) stage-two linear regression model (S2LR). The goal of the first stage is to distinguish between subjects with normal and abnormal LC-based sleep tests. The second stage of the algorithm uses linear regression to identify the actual AHI of the people identified to have sleep apnea by the S1DT.

The linear regression model was trained on only mild, moderate, or severe sleep apnea data sets. This linear regression model was used to predict AHI values. We found linear correlation between the extracted features and the AHI score in the available data. Therefore a more complex model was not necessary to process our data. However, more sophisticated machine learning methods might be useful when processing data collected from a larger study. The predicted AHI output of the linear regression model was subsequently used to classify the OSA severity as either mild or moderate/severe. Sleep tests with estimated AHI values between 5–15 events/h were assigned to the mild category, whereas sleep tests with estimate AHI values greater than 15 events/h were placed into the moderate/severe category [21]. Cases of moderate and severe OSA were considered a single class due to the fact that both severity levels are clinically managed using similar therapeutic approaches, CPAP being the gold standard treatment [7,8,9,10]. The following equation defines the multiple linear regression model optimized for AHI estimation where X[2], X[3], and X[5] are defined in Table 1:

(1) $$\underset{predicted}{AHI}=11.94+23.06X{\left[2\right]}^{2}+8.74X\left[3\right]-15.04X\left[5\right].$$

Since we obtained data from the LC sensors across multiple nights for most subjects, we selected the highest predicted AHI across both nights as the estimated AHI. It is known that there is high night-to-night variability of OSA and there is a tendency for physicians to err on the side of higher AHI estimations.

We also investigated whether the AHI estimation was influenced by the use of the HSAT equipment worn by the participants. We recorded LC signals corresponding to one or two additional nights in which subjects were sleeping at home and were not monitored with the HSAT system. When analyzing the LC signals recorded concurrently with a gold standard method, we excluded the portions of the recordings marked as invalid by the clinician who scored the studies based on the gold standard signals. Since we did not have a gold standard for those additional nights in which the participants were only monitored using our developed sensing device, we excluded the portions of the recorded signals that were not suited for AHI estimation by using a windowed approach, whereby overlapping 3-h recording segments were processed using our machine learning models. For each segment, we estimate the AHI and the resulting AHI time series was processed using a rolling median filter to detect and remove AHI outliers. Values with large deviation from the median of the time-ordered AHI sequence might indicate the presence of noise in the evaluated portion of the study and were therefore removed. The final AHI assigned to the study was calculated as the average of the remaining AHI values after outlier removal. We selected a 3-h window size since a minimum of 2- to 4-h recording is recommended by AASM when testing for OSA using in-laboratory PSG and HSAT [20].

## 3. Results

**Figure 6:**
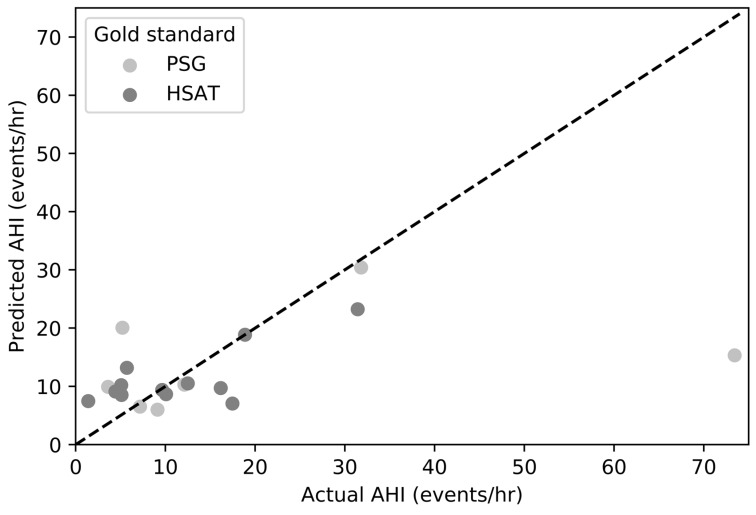
Actual AHI versus predicted AHI. Pearson’s R = 0.47 (polysomnography (PSG) vs. LC: R = 0.37, home sleep apnea test (HSAT) vs. LC: R = 0.74).

We evaluated our prediction models on 13 PSG recordings corresponding to a single in-clinic sleep test and 22 HSAT recordings corresponding to one or two overnight sleep studies per subject (see Figure 1). The average duration of interpretable portions of PSG studies was 5.73 ± 2.15 h per night whereas the average duration of interpretable portions of HSAT studies was 5.37 ± 2.82 h per night. Table 2 and Table 3 show the results for the categorization of sleep apnea severity and the estimated AHI values for patients who tested positive for sleep apnea during PSG and HSAT studies, respectively. Based on the results from all individual overnight sleep studies, including studies carried out in the sleep clinic and at patients’ homes, S1DT reached an overall OSA detection accuracy of 82.9% with sensitivity of 88.9% and specificity of 76.5%. The positive predictive value (PPV) and negative predictive value (NPV) are 80.0% and 86.7%, respectively. When the accuracy analysis was performed for studies carried out at the sleep clinic and at home separately, the S1DT correct detection rate is calculated to be 69.2% for in-clinic studies (with sensitivity = 75.0%, specificity = 60.0%, PPV = 75.0%, and NPV = 60.0%), and 90.9% for in-home studies (with sensitivity = 100.0%, specificity = 83.3%, PPV = 83.3%, and NPV = 100.0%). The final OSA severity classification accuracy evaluated using the combined predictions from S1DT and S2LR was calculated to be 74.3%, corresponding to 61.5% and 81.8% correctly classified in-clinic and in-home tests, respectively, with underline median absolute AHI error of 4.59 events/h. The accuracy difference in OSA diagnosis between in-clinic and in-home studies might be explained by the amount of data available for the estimation of final OSA diagnosis since a single night of data was available for in-clinic studies while generally two nights were used for in-home OSA severity classification. The average AHI estimated by the sleep clinician from manual PSG and HSAT scoring across all subjects and nights was 14.25 ± 8.70 events/h. The overall correlation between estimated AHI and reference AHI values was assessed using the Pearson’s correlation coefficient, which was calculated to be 0.47 (Figure 6). This correlation is affected by the large estimation error on a single sleep test whose true AHI score is larger than the 3-sigma cutoff value AHI = 40.35 events/h. Figure 5 shows the extreme data point at actual AHI = 73.43 (predicted AHI = 15.32).

We also investigated the effect of wearing HSAT sensors versus not wearing HSAT sensors on the estimated OSA severity. We compared the estimated AHI values obtained during two nights where patients were monitored simultaneously by HSAT and LC equipment (Table 3) against AHI obtained from LC recordings collected during two night in which subjects did not wear HSAT sensors (Table 4). We found that when participants wore the HSAT equipment while sleeping, it did not have a significant effect in AHI estimation. When we compared the AHI estimation for the two nights when participants wore the HSAT equipment with the two nights when they did not wear the HSAT equipment, we found that there was no statistically significant difference in the AHI estimation (*t*-value = −0.51, *p*-value = 0.62). Table 5 shows results of the survey given to the participants. Most of the participants found the system easy to install and found that the bed felt stable and comfortable while the LC system was installed.

## 4. Discussion

**Figure 7:**
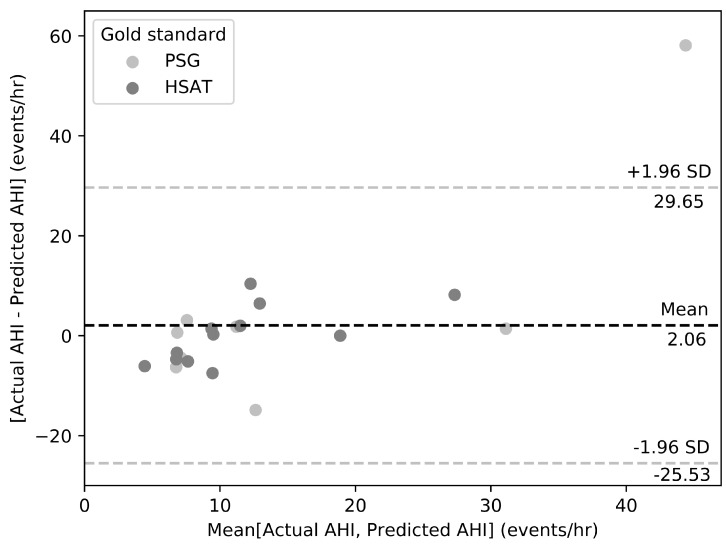
Bland–Altman plot for assessing agreement between LCs AHI and manual scoring of gold standard PSG and HSAT studies.

This study demonstrates the feasibility of diagnosing OSA using an easy-to-install pressure sensing device coupled with signal processing and machine learning algorithms that automatically process pressure signals and classify OSA severity in unattended settings, either in the sleep clinic or at the patient’s home. Using the PSG in the sleep clinic and Type 3 HSAT systems in the home as reference, our LC-based system had a high OSA detection accuracy. The Bland–Altman plot in Figure 7 shows that the prediction algorithm had a bias of 2.06 events/h and an agreement range from −25.53 to 29.69 events/h, with most AHI differences falling within the range −12.41 to 10.64 events/h. The bias and agreement bands are influenced by the large error in the estimation of the AHI for a single subject from a PSG test conducted in the sleep clinic whose actual AHI is beyond the 3-sigma range as discussed under the Results section. The underestimation of AHI for the severe subject with an AHI of 73.43 events/h was likely caused by the distribution of AHI scores within the small cohort of participants in the study. Despite this large AHI estimation error, that PSG study was accurately estimated to be in the moderate/severe category by our method. A more robust approach for an accurate AHI estimation would have been the detection and counting of individual apneas and hypopneas, which is still a challenging engineering problem that can be solved using more sophisticated machine learning models that require large amounts of data annotated by multiple specialized sleep clinicians to account for inter-rater variability.

**Figure 8:**
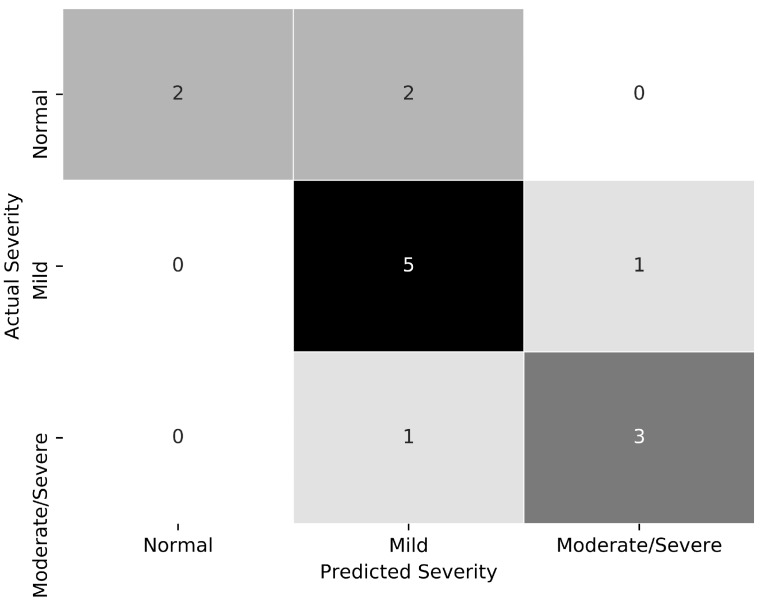
Confusion matrix for OSA severity classification from multiple nights, including in-clinic and in-home studies, with reference AHI.

Although one night of sleep testing is the standard for diagnosis of OSA, the accuracy of OSA detection with only one-night of testing requires high sensitivity of the test method and a stable disease of interest to warrant a low rate of false-negative tests [22,23]. For people with OSA, there can be a large night-to-night variability, including extreme OSA on one night and normal (i.e., very little OSA) another night. This night-to-night variability may be due to the subject’s lying position or the duration of rapid-eye movement (REM) during sleep. In practice, one night supine can provide completely different test results than one night lateral for some subjects. Therefore, monitoring a patient’s sleep over multiple nights might provide a more sensitive OSA detection test. In this study, using the available data collected during multiple nights (including LC signals obtained in the sleep clinic and at home), and selecting the most severe night as the final OSA severity, we found that all subjects with OSA were correctly identified by our proposed LC-based system (sensitivity = 100.0%). However, our method tends to overestimate the severity of OSA (specificity = 50.0%) in accordance with the confusion matrix in Figure 8.

The diagnosis of sleep apnea is given for an AHI > 5. Mild to moderate OSA is indicated for an AHI of 5 to 14 yet sensitivity and specificity of our gold standards (PSG and HSAT) used in this study break down when different approved scoring criteria are used or when comparing home sleep apnea testing to PSG. For the in-home portion of the study, the LC-based system tended to over-estimate AHI compared with the HSAT system. It is important to consider that the HSAT system did not include EEG sensors that are typically used to obtain a more accurate AHI score. In the future, we will further investigate whether the overestimation of OSA by our sensor system still occurs when we use a more accurate gold standard method for obtaining AHI that includes EEG within the HSAT system. We found that OSA severity overestimation was more prevalent when comparing LC against HSAT results compared with the OSA PSG done in the lab. Other groups have also found that HSAT systems tend to underestimate sleep apnea severity and provide false negative results for some patients with mild sleep apnea when compared to PSG [24,25,26]. Additional work on this finding is needed to confirm or reject the hypothesis that LC can capture other physiological signs associated with distorted breathing. Future work assessing our false positives with instrumentation more sensitive than gold standard PSG may prove useful to confirm or refute the absence of a meaningful physiologic change at those times. For example, PSG relies on human visual scoring for detection of hypopnea, such as a 3-s visually detected change in frequency to score arousal and a 30% reduction of an uncalibrated flow excursion with a variable baseline, both of which are used for gold standard determination of hypopnea. Other known more sensitive tools, such as computer-assisted electroencephalogram analysis or esophageal manometry, may or may not identify associations with meaningful physiologic changes at times we classified as false positives.

## 5. Conclusions

The results of this pilot study showed the potential of a LC-based system to monitor sleep disorders within the home under free-living conditions. The accuracy results of the system show that we can identify patients with OSA without subject contact or maintenance of sensors with an accuracy of 82.9% (sensitivity = 88.9%, specificity = 76.5%). Furthermore, we were able to demonstrate LC deployability in the home environment, as indicated by the high ratings given by patients regarding several aspects of the device deployment, including ease of installation, bed stability, and effect of the installed sensor on the feel of the bed. Given the results of this pilot study, additional multiple-night studies with a larger sample size should be done to better characterize the accuracy, sensitivity, and specificity of our method over time, correcting for limitations in the gold standard PSG, including the first night effect and night-to-night variability in syndrome severity.

There are several advantages of the evaluated system over full PSG and HSAT devices. Our non-contact sensor has a good sensitivity and does not require battery changes, maintenance or sensor placement providing patients with an unobtrusive and easy-to-install sleep monitoring device that continuously monitors patients’ sleep patterns, breathing, and automatically estimates the patient’s OSA severity. The system evaluated in this study is cost-effective and can potentially benefit the healthcare system since more patients might be diagnosed early, leading to substantial economical savings through the prevention of serious health complications associated with untreated OSA. Additional applications include long-term non-contact monitoring of sleep and breathing in pertinent populations over time, such as military or pharmacologic trials.

Limitations of the study include the small sample size and the bias introduced by the gender and age imbalance of the participants in the pilot study. However, despite the small sample size, we were able to achieve good accuracy of the AHI estimation algorithm. We expect that results from this preliminary study will help to guide further evaluations of LC-based sleep apnea testing within homes and clinics.

## Figures and Tables

**Table 1:** Spectral features used for OSA severity classification. S1DT = stage-one decision tree; S2LR = stage-two linear regression model.

Feature	Frequency Sub-Band	Statistic Calculated from Fast Fourier Transform (FFT) Coefficients	Classification Stage
X[1]	0.06–0.08 Hz	kurtosis	S1DT
X[2]	0.36–0.38 Hz	skewness	S2LR
X[3]	0.96–0.98 Hz	skewness	S2LR
X[4]	1.18–1.20 Hz	kurtosis	S1DT
X[5]	1.40–1.42 Hz	kurtosis	S2LR
X[6]	1.68–1.70 Hz	skewness	S1DT

**Table 2:** Results of in-clinic overnight attended polysomnogram.

Patient ID	Study Duration	Clinical Category		AHI		Severity	
	(hours)	Actual	Predicted	Actual	Predicted	Actual	Predicted
1	7.10	Abnormal	Abnormal	7.16	6.53	Mild	Mild
2	0.00	-	-	-	-	-	-
3	1.15	Abnormal	Abnormal	31.81	30.40	Severe	Moderate/Severe
4	6.34	Abnormal	Abnormal	9.12	6.01	Mild	Mild
5	1.12	Abnormal	Abnormal	73.43	15.32	Severe	Moderate/Severe
6	7.33	Normal	Abnormal	3.60	9.92	Normal	Mild
7	5.16	Abnormal	Normal	5.34	<5	Mild	Normal
8	7.04	Normal	Abnormal	4.89	9.37	Normal	Mild
9	5.65	Normal	Normal	3.06	<5	Normal	Normal
10	6.93	Normal	Normal	2.20	<5	Normal	Normal
11	6.21	Abnormal	Abnormal	12.11	10.30	Mild	Mild
12	7.07	Abnormal	Abnormal	5.20	20.06	Mild	Moderate/Severe
13	6.01	Abnormal	Normal	7.06	<5	Mild	Normal
14	7.39	Normal	Normal	0.83	<5	Normal	Normal

**Table 3:** In-home unattended sleep apnea testing using HSAT + LCs.

Patient ID	Night	Study Duration	Clinical Category		AHI		Severity	
		(hours)	Actual	Predicted	Actual	Predicted	Actual	Predicted
1	1	7.76	Abnormal	Abnormal	9.63	9.38	Mild	Mild
	2	9.08	Abnormal	Abnormal	10.07	8.66		
2	1	6.93	Abnormal	Abnormal	17.46	7.05	Moderate	Mild
	2	5.35	Abnormal	Abnormal	16.16	9.71		
3	1	3.56	Abnormal	Abnormal	12.49	10.51	Moderate	Moderate/Severe
4	1	8.15	Normal	Normal	3.61	<5	Normal	Normal
	2	5.37	Normal	Normal	1.51	<5		
5	1	4.83	Abnormal	Abnormal	31.42	23.23	Severe	Moderate/Severe
6	1	5.07	Normal	Abnormal	4.42	9.12	Normal	Mild
	2	0.77	Normal	Normal	1.34	<5		
7	1	5.00	Abnormal	Abnormal	5.70	13.19	Mild	Mild
	2	1.40	Abnormal	Abnormal	5.07	10.20		
8	1	8.57	Abnormal	Abnormal	5.11	8.53	Mild	Mild
9	1	1.54	Normal	Normal	4.02	<5	Normal	Mild
	2	1.45	Normal	Abnormal	1.40	7.48		
10	1	9.59	Normal	Normal	1.49	<5	Normal	Normal
	2	9.05	Normal	Normal	2.14	<5		
11	1	3.03	Normal	Normal	1.67	<5	Normal	Normal
12	1	2.82	Normal	Normal	2.17	<5	Normal	Normal
13	1	4.26	Abnormal	Abnormal	18.87	18.87	Moderate	Moderate/Severe
14	1	8.70	Normal	Normal	0.71	<5	Normal	Normal
	2	5.88	Normal	Normal	0.18	<5		

**Table 4:** Estimated AHI when monitoring patients at home with LCs device only.

Patient ID	Night	Clinical Category	AHI	Severity
		Predicted	Predicted	Predicted
1	1	Abnormal	14.08	Mild
	2	Abnormal	9.47	
2	1	Abnormal	10.38	Mild
	2	Abnormal	11.52	
3	1	Abnormal	6.94	Mild
	2	Normal	<5	
4	1	Abnormal	5.61	Mild
	2	Normal	<5	
5	1	Abnormal	8.55	Mild
	2	Abnormal	14.72	
6	1	Normal	<5	Mild
	2	Abnormal	6.63	
8	1	Abnormal	13.07	Mild
	2	Normal	<5	
9	1	Normal	<5	Normal
	2	Normal	<5	
10	1	Normal	<5	Mild
	2	Abnormal	5.27	
12	1	Abnormal	24.84	Severe
	2	Normal	<5	
13	1	Normal	<5	Mild
	2	Abnormal	9.00	
14	1	Normal	<5	Normal
	2	Normal	<5	

**Table 5:** LCs deployability and comfort survey results. Most respondents found the system easy to install and did not notice any problems with stability or the comfort of the bed.

Rating *	Ease of installation	Stability	Comfort
1	0.00%	0.00%	0.00%
2	7.14%	0.00%	0.00%
3	7.14%	0.00%	0.00%
4	28.57%	14.29%	14.29%
5	57.14%	85.71%	85.71%

* 5 is the best possible rating.
